# Temporal variability of diazotroph community composition in the upwelling region off NW Iberia

**DOI:** 10.1038/s41598-019-39586-4

**Published:** 2019-03-06

**Authors:** Víctor Moreira-Coello, Beatriz Mouriño-Carballido, Emilio Marañón, Ana Fernández-Carrera, Antonio Bode, Eva Sintes, Jonathan P. Zehr, Kendra Turk-Kubo, Marta M. Varela

**Affiliations:** 1 Departamento de Ecoloxía e Bioloxía Animal, Universidade de Vigo, Vigo, Spain; 2 Instituto Español de Oceanografía, A Coruña, Spain; 3 Instituto Español de Oceanografía, Baleares, Spain; 4 Ocean Sciences Department, University of California, Santa Cruz, California USA

## Abstract

Knowledge of the ecology of N_2_-fixing (diazotrophic) plankton is mainly limited to oligotrophic (sub)tropical oceans. However, diazotrophs are widely distributed and active throughout the global ocean. Likewise, relatively little is known about the temporal dynamics of diazotrophs in productive areas. Between February 2014 and December 2015, we carried out 9 one-day samplings in the temperate northwestern Iberian upwelling system to investigate the temporal and vertical variability of the diazotrophic community and its relationship with hydrodynamic forcing. In downwelling conditions, characterized by deeper mixed layers and a homogeneous water column, non-cyanobacterial diazotrophs belonging mainly to *nifH* clusters 1G (Gammaproteobacteria) and 3 (putative anaerobes) dominated the diazotrophic community. In upwelling and relaxation conditions, affected by enhanced vertical stratification and hydrographic variability, the community was more heterogeneous vertically but less diverse, with prevalence of UCYN-A (unicellular cyanobacteria, subcluster 1B) and non-cyanobacterial diazotrophs from clusters 1G and 3. Oligotyping analysis of UCYN-A phylotype showed that UCYN-A2 sublineage was the most abundant (74%), followed by UCYN-A1 (23%) and UCYN-A4 (2%). UCYN-A1 oligotypes exhibited relatively low frequencies during the three hydrographic conditions, whereas UCYN-A2 showed higher abundances during upwelling and relaxation. Our findings show the presence of a diverse and temporally variable diazotrophic community driven by hydrodynamic forcing in an upwelling system.

## Introduction

Diazotrophs are a small and diverse group of microorganisms (comprising Bacteria and Archaea) that can use the large dinitrogen (N_2_) inventory in aquatic and terrestrial ecosystems by the energetically-costly process of biological N_2_ fixation (BNF)^1^. These microbes possess the *nifH* gene that encodes the highly conserved iron-protein subunit of the nitrogenase enzyme, responsible for catalyzing the reduction of atmospheric N_2_ to bioavailable ammonium (NH_4_^+^)^2^. BNF has fundamental biogeochemical significance, particularly in oligotrophic regions, because it fertilizes the ocean by supplying new nitrogen (N).

There is a large diversity of marine diazotrophs and their wide distribution^3–5^ spans the range of marine environments where BNF is a relevant process^6^. Cyanobacterial diazotrophs have traditionally been considered the most important oceanic N_2_-fixing organisms. Besides the most studied diazotrophic cyanobacteria, i.e. the filamentous *Trichodesmium*^7,8^, symbionts of diatoms such as *Richelia intracellularis*^9^ and the unicellular free-living *Crocosphaera* sp. or UCYN-B^10^, marine diazotrophs also comprise other unicellular cyanobacteria (the uncultivated group A or UCYN-A and the presumably free-living UCYN-C, which contains cultivated cyanobacteria such as *Cyanothece* sp.), and non-cyanobacterial diazotrophs (heterotrophic Bacteria and Archaea)^4,11^. Heterotrophic bacterial diazotrophs include a large diversity of phylogenetic groups such as Proteobacteria^4^.

The cyanobacterium UCYN-A (or *Candidatus* Atelocyanobacterium thalassa) is an important member of the global diazotroph community due to its broad distribution in marine environments and relatively high BNF rates^12,13^. It lives in association with a picoeukaryotic prymnesiophyte^14,15^, and the single-celled symbiosis established between them is a potential model for observing the evolution of organelles in eukaryotic cells, specifically N_2_-fixing organelles^16^. Despite the ecological, biogeochemical and evolutionary relevance of UCYN-A, its low abundance in the marine environment makes its identification and quantification challenging. Regardless, a targeted approach recently adapted by Turk-Kubo *et al*.^17^ to define UCYN-A oligotypes, finely resolved taxonomic groups based on nucleotide positions with high variability^18^, has facilitated progress in the study of its diversity and ecology. Six UCYN-A sublineages (UCYN-A1 to -A6) have previously been defined based on the genetic diversity of *nifH* sequences available in the NCBI GenBank database^13,15,17^. Recent findings indicate that each of these sublineages occupy different ecological niches^13,17^.

Diazotroph communities from contrasting marine environments along the global ocean differ markedly in their composition^4,19,20^. Although studies concerning diazotrophic composition are not directly comparable due to different methodological approaches used, *nifH* clone libraries from open ocean show in general lower richness and diversity than libraries from the dynamic estuarine and coastal regions^4,19,21–23^. The higher richness and diversity of diazotrophs found in coastal marine environments may be explained by different factors, such as the allochthonous supply of diazotrophs of riverine origin^24,25^.

Despite the high dynamic nature of diazotroph communities^26^, the insight on the temporal variability in response to hydrographic and environmental changes is limited^27–30^. Therefore, the factors controlling the diversity, abundance and N_2_-fixing activity of diazotrophs are not well defined^6,31–34^. Furthermore, most studies have been conducted in the warm waters of the (sub)tropical region of the western basin of the North Atlantic Ocean (80°–40°W), where *Trichodesmium* dominates the community^12,35,36^. Hence, little is known about the temporal and spatial distribution of the diazotroph community in cooler, temperate (latitude > 40°) waters such as the nutrient-rich northwest Iberian upwelling system.

The upwelling region of the northwestern Iberian Peninsula is one of the eastern boundary upwelling ecosystems in the global ocean^37^. It is seasonally influenced by wind-driven upwelling (from April to September) and downwelling (from October to March) events^38^. The frequent nutrient inputs, along with their amplification due to remineralisation inside the Rías^39,40^, fuel the primary production sustaining a rich marine ecosystem^38,41^. Therefore, in this system, high-frequency variability dominates physical, chemical and biological factors^42–44^. Knowledge of the presence and activity of diazotrophs in this N-rich temperate region has been limited to the observation carried out in summer 2009 by Agawin *et al*.^45^ and Benavides *et al*.^46^, who described relatively low BNF rates, mainly attributed to UCYN-A.

Herein, we describe the diazotroph community composition (based on the nitrogenase gene, *nifH*) of the upwelling region off northwestern Iberia between February 2014 and December 2015. Our main goal was to investigate the temporal and vertical variability of the diazotroph community composition, and its relationship with the hydrodynamic forcing in productive waters of an upwelling region.

## Results

We amplified and sequenced the *nifH* gene in 24 samples collected in the upper 70 m between February 2014 and December 2015 during 9 one-day samplings carried out in the temperate upwelling region of the northwestern Iberian Peninsula (more details in Materials and Methods).

### Diazotroph community composition

A total of 581732 *nifH* sequences were obtained, which clustered into 1276 OTUs at 92% nucleotide identity. The total number of sequences per sample ranged from 9534 in May 2014 (40 m) to 49612 in July 2015 (0 m) (Table S1). However, a subsampling to the minimum sequencing depth (9534 sequences) was done to allow for beta diversity comparisons between samples. Rarefaction (Fig. S1) and species accumulation (Fig. S2) curves, as well as the small differences found between the Chao1 richness estimator and the number of OTUs observed (Fig. S3), suggest that the sampling effort (number of sequences sampled) and the *nifH* library obtained were appropriate to cover and reliably characterize the overall *nifH* diversity in the region.

Overall, the *nifH* analysis revealed that all sequences belonged to unicellular cyanobacterial and non-cyanobacterial diazotrophs. The most abundant *nifH* clusters were 3 (mainly putative anaerobes, 664 OTUs), 1G (which is comprised of mainly Gammaproteobacteria, 285 OTUs) and 1B (Cyanobacteria, 74 OTUs), which represented 36%, 28% and 21% of total sequences, respectively. We compared by BLASTn^47^ the representative sequences of Gammaproteobacterial OTUs with the sequence of the globally distributed Gammaproteobacteria-affiliated phylotype γ-24774A11 (EU052413 accession number)^48^. However, we did not recover this sequence type, and Gammaproteobacterial OTUs were at most 80–82% similar at the nucleotide level. The other *nifH* clusters, including 253 OTUs, each accounted for less than 8% of the total relative abundance. The diazotroph community composition in this region was uneven since few OTUs dominated (Fig. S4). Most *nifH* sequences belonged to OTUs affiliated with UCYN-A (subcluster 1B, 21% of total sequences clustered mostly in OTUs 0, 2854 and 338 with 100% nucleotide identity to CP001842), *Pseudomonas stutzeri* (subcluster 1G, Genbank accession number CP002622) as the closest cultivated relative (21% of total sequences clustered mainly in 10 OTUs, with 93–96% identity to *P. stutzeri*), *Opitutaceae* bacterium (cluster 3, 9% of total sequences clustered principally in OTU 1 with 94% identity to CP007053) and *Desulfovibrio aespoeensis* (cluster 3, 8% of total sequences clustered mainly in OTU 2 with 95% identity to CP002431). Whereas the rest of diazotrophs represented <7% each.

**Figure 1:**
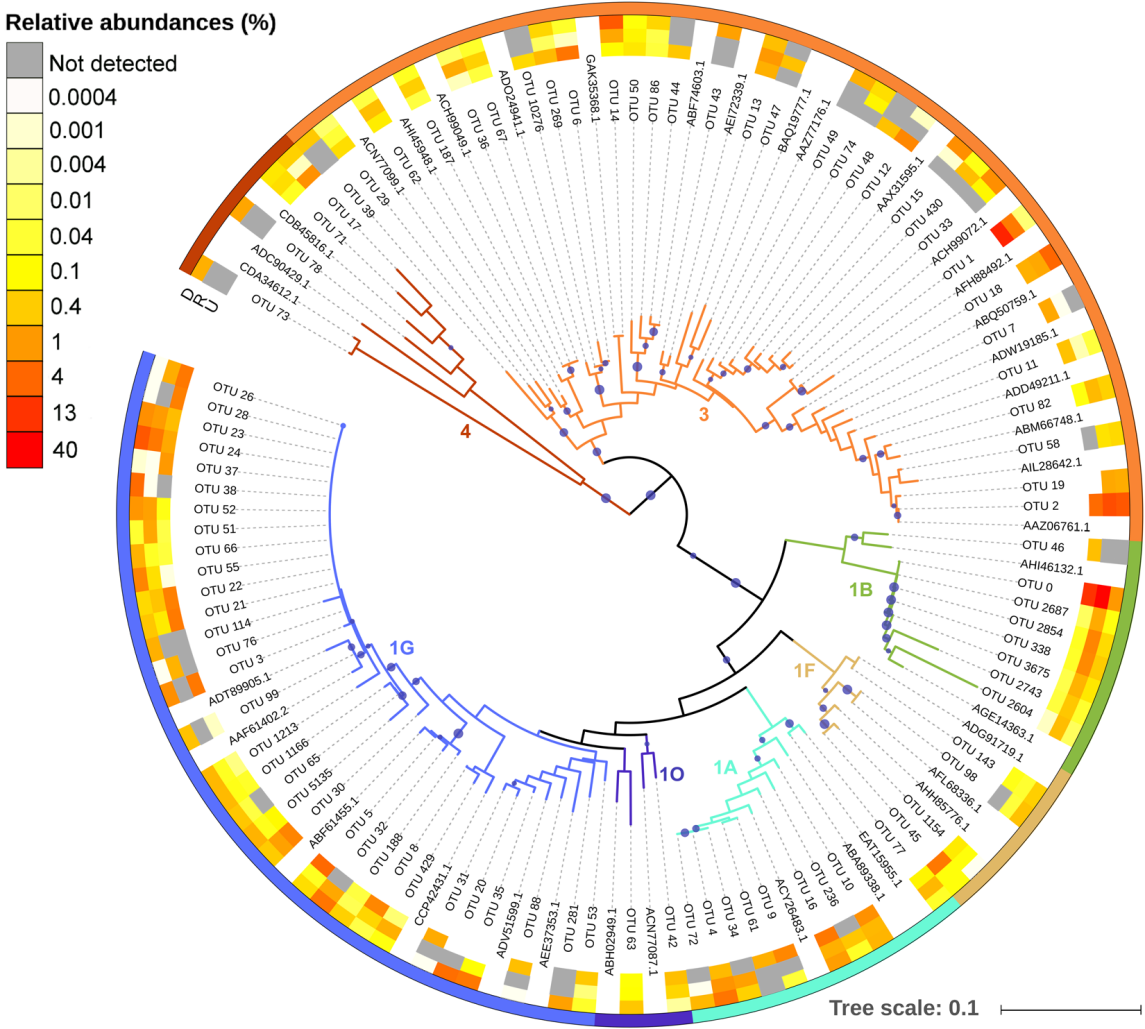
Neighbor-joining phylogenetic tree of the 90 most abundant OTUs in the NGS library (>700 sequences each, representing 83.1% of the dataset) at 92% translated amino acid *nifH* sequence identity and their closest cultivated relatives (based on BLASTx). Canonical *nifH* clusters affiliation according to the convention defined by Zehr *et al*.^4^ are indicated by coloured branches and external strip. The heatmap associated shows the relative abundance in different conditions (D, downwelling; U, upwelling; and R, relaxation) for each OTU, in which grey colour indicates no sequences detected within the OTU cluster. Accession numbers for reference sequences are indicated on the tree. Bootstrap support values (≥50%) for 1000 replicate trees are displayed with size proportional blue circles next to the nodes.

We used the 90 most abundant OTUs, which accounted for 83.1% of the *nifH* sequences, to construct the phylogenetic tree of the diazotroph community present in the region (Fig. 1). Most OTUs included in the tree (a total of 82 OTUs) belonged to *nifH* subclusters from non-cyanobacterial diazotrophs. However, although cyanobacterial OTUs were less diverse (subcluster 1B, 8 OTUs), they were very abundant, comprising a large number of sequences. Branches of cyanobacterial OTUs belonged mostly to UCYN-A, except OTU 46 which was related to *Nostoc punctiforme* (92% nucleotide identity to the best blast hit with Genbank accession number CP001037) and was only detected in surface waters during the upwelling conditions sampled in June 2015. UCYN-A OTUs were mainly represented by OTU 0 with 100% identity to CP001842. But they also included 4 UCYN-A1 OTUs (OTUs 2687, 2854, 338 and 3675), OTU 2743 and OTU 2604 with 100%, 94% and 92% identity to CP001842, respectively. OTU 0 was by far the most abundant OTU, representing 18% of the *nifH* library. OTUs affiliated with subcluster 1G were related mostly to *P. stutzeri* (92–96% identity to CP002622). OTU 1 (related with *Opitutaceae* bacterium), OTU 2 (related to *Desulfovibrio aespoeensis*) and OTU 24 (related with *P. stutzeri*) were the most abundant non-cyanobacterial OTUs, accounting for 9%, 6% and 3% of total *nifH* sequences, respectively.

### Temporal and vertical variability of the diazotroph community composition

**Figure 2:**
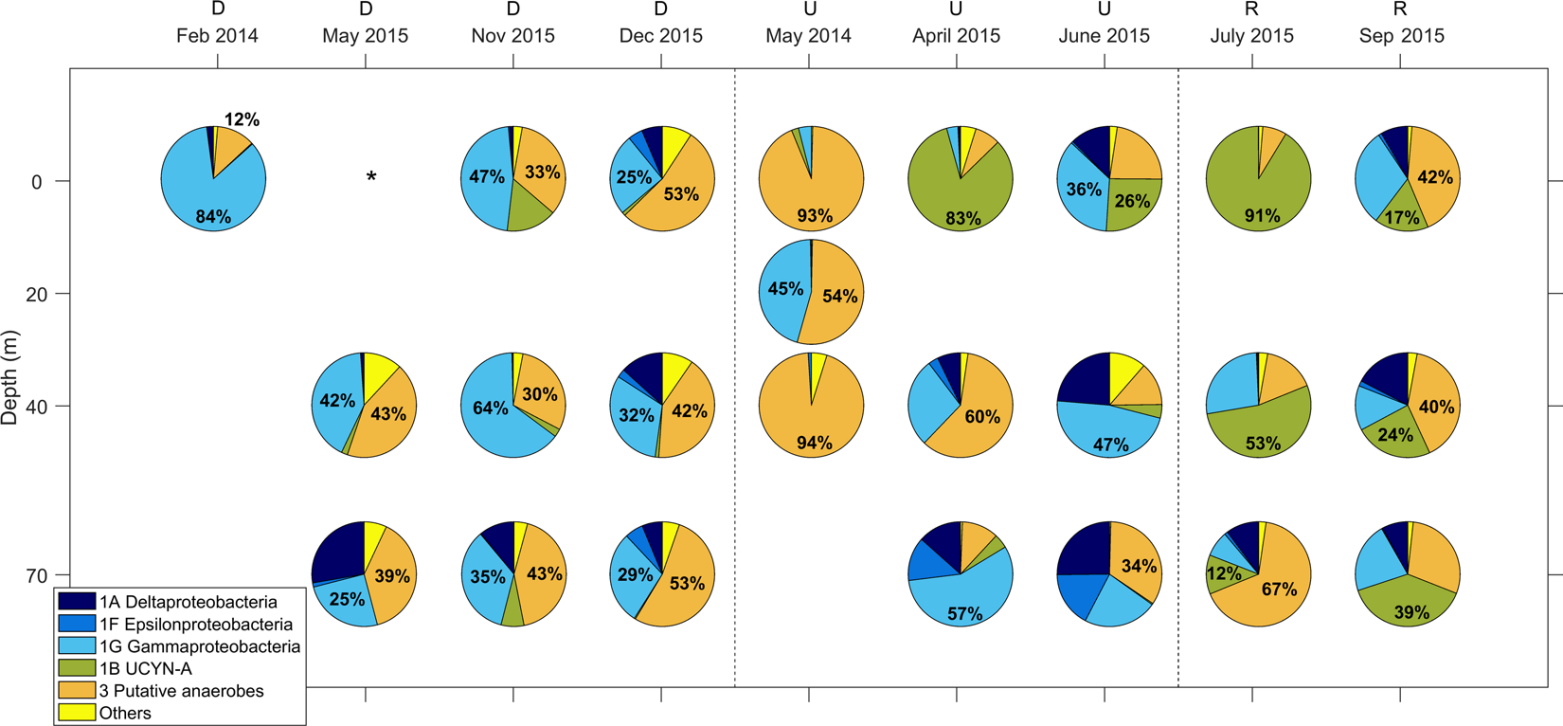
Distribution of the most abundant *nifH* clusters in the NGS library (representing > 2% of total *nifH* sequences). Phylogenetic assignments of the clusters are also indicated. ‘Others’ includes mainly *nifH* cluster 4. Upper characters indicate the hydrographic condition during each sampling date (D, downwelling; U, upwelling; and R, relaxation). *nifH* gene was not detected by PCR amplification in the sample from May 2015 (0 m) (indicated by asterisk). Number of sequences obtained per sample after quality filtering are shown in Table S1.

Diazotroph community composition in this region exhibited temporal and vertical variability (Fig. 2). In general, upwelling conditions were dominated (based on relative abundance) by non-cyanobacterial diazotrophs from subcluster 1G (Gammaproteobacteria), mainly *P. stutzeri*-like sequences from OTU 24, and cluster 3 (putative anaerobes). The only exceptions were the surface samples of April and June 2015, where Cyanobacteria, represented mostly by UCYN-A of OTU 0, had high relative abundances (83% and 26%, respectively). Upwelling samples from May 2014 reflected a large divergence in their composition. They were characterized by a clear predominance (always > 54%) of OTU 1, which are associated with cluster 3, mainly *Opitutaceae* bacterium-like taxa, which were almost absent in the rest of samples.

All depths during relaxation were characterized by increased relative abundance of subcluster 1B (Cyanobacteria) (ranging from 12% to 91%), mainly UCYN-A sequences clustered in OTU 0. Non-cyanobacterial diazotrophs were represented mostly by cluster 3 (*Desulfovibrio*-like sequences clustered mainly in OTU 2) and 1G. After OTU 0, OTU 2 was the most relevant cluster during relaxation.

During downwelling, diazotroph community was dominated by non-cyanobacterial diazotrophs at all depths. This group was mostly represented by *nifH* subcluster 1G (always > 25%), mainly *P. stutzeri*-like sequences, and cluster 3 (always > 12%). In any case, no OTU dominated clearly, leading to the high *nifH* diversity observed during this hydrographic regime. During downwelling conditions, the mean number of OTUs observed (248 ± 82 OTUs), Chao1 richness (306 ± 127 OTUs) and Shannon diversity (3.6 ± 0.5) were significantly higher (Bonferroni comparisons, *p* < 0.05) than during upwelling (103 ± 25 OTUs, 115 ± 25 OTUs and 2.0 ± 1.0, respectively), and slightly higher than during relaxation (176 ± 74 OTUs, 200 ± 86 OTUs and 2.9 ± 0.9, respectively) (Fig. S3).

**Figure 3:**
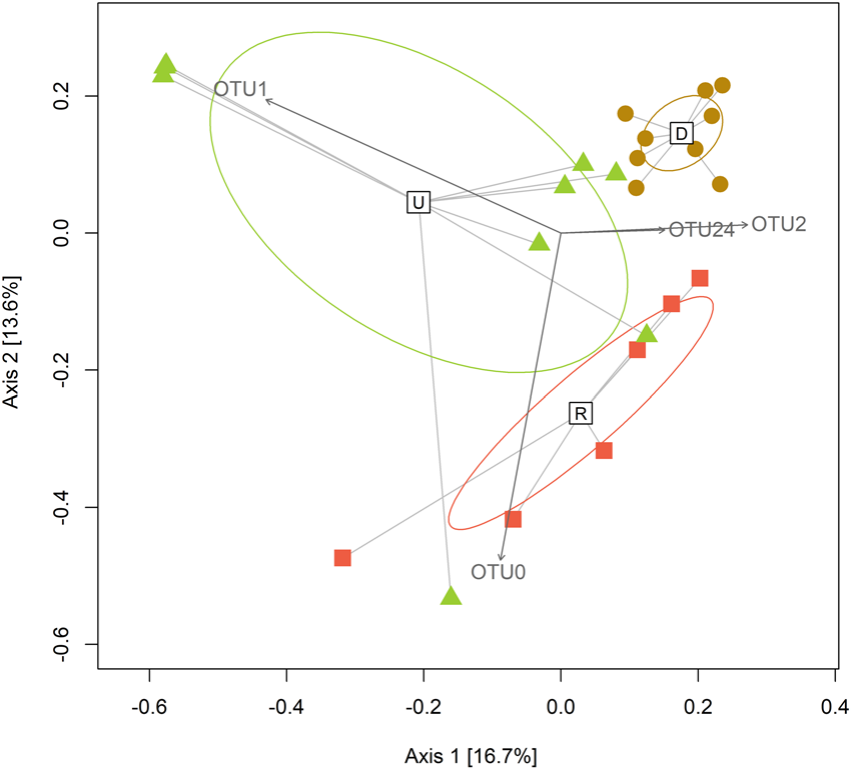
Principal Coordinates Analysis (PCoA) based on Bray-Curtis dissimilarity distance matrix illustrating the *nifH* compositional differences between samples from downwelling (brown circles), upwelling (green triangles) and relaxation (red squares). Sample group centroids for each hydrographic condition and confidence ellipses displaying the standard deviation of centroid locations are also represented. According to SIMPER analysis, the OTUs that contributed most to the compositional differences observed between hydrographic conditions were fitted through significant (*p* < 0.05) vector overlays onto the PCoA ordination. OTU 0 (UCYN-A, 100% nucleotide identity to Genbank accession number CP001842), OTU 1 (cluster 3, Opitutaceae bacterium-like taxa, 94% identity to CP007053), OTU 2 (cluster 3, *Desulfovibrio aespoeensis*-like, 95% identity to CP002431), and OTU 24 (subcluster 1 G, *Pseudomonas stutzeri*-like, 94% identity to CP002622).

The temporal and vertical variability observed in the composition of the diazotrophic community is illustrated by the Principal Coordinates Analysis (PCoA) (Fig. 3) based on Bray-Curtis dissimilarities (Table S2). A higher phylogenetic similarity was observed in the samples collected during downwelling compared to upwelling and relaxation, which exhibited larger variation in their composition, mainly during upwelling, when a higher vertical variability was observed. During downwelling, the water column was characterized by homogeneous conditions and deep mixed layers (Fig. S5). Upwelling and relaxation periods were characterized by relatively enhanced vertical stratification and a larger vertical variability in hydrographic conditions. Significant differences in *nifH* composition were observed between the three hydrographic conditions (PERMANOVA, *p* < 0.001). Specifically, community composition during downwelling was significantly different from those observed during upwelling and relaxation (Bonferroni comparisons, *p* < 0.05), whereas no significant differences were observed between upwelling and relaxation. As revealed by the SIMPER analysis, the compositional differences observed were mainly due to the relative contribution of the most abundant OTUs: OTU 0 (explained between 7% and 20% of variability, UCYN-A), OTU 1 (11–12%, *Opitutaceae* bacterium-like taxa), OTU 2 (3–4%, *Desulfovibrio aespoeensis*-like taxa) and OTU 24 (~3%, *P. stutzeri*-like taxa). OTU 0 correlated significantly (*r*^2^ = 0.906, *p* < 0.001), as indicated by the direction and length of its vector overlay onto PCoA, with the samples collected in relaxation and surface samples of April and June 2015 (upwelling), which were characterized by the predominance of this cluster (Fig. 2). Vector overlay of OTU 1 showed a strong correlation (*r*^2^ = 0.859, *p* < 0.001) with the upwelling samples from May 2014 (located in upper left corner of PCoA), which were dominated by this putative anaerobe cluster. OTU 2 was a relevant cluster both in downwelling and relaxation, as shown by its vector (*r*^2^ = 0.286, *p* < 0.05). Finally, OTU 24 was very abundant in downwelling but it was also present in upwelling and relaxation samples. Thus, the correlation of its vector with downwelling samples was weaker (*r*^2^ = 0.088, *p* < 0.05).

### UCYN-A oligotypes diversity

**Figure 4:**
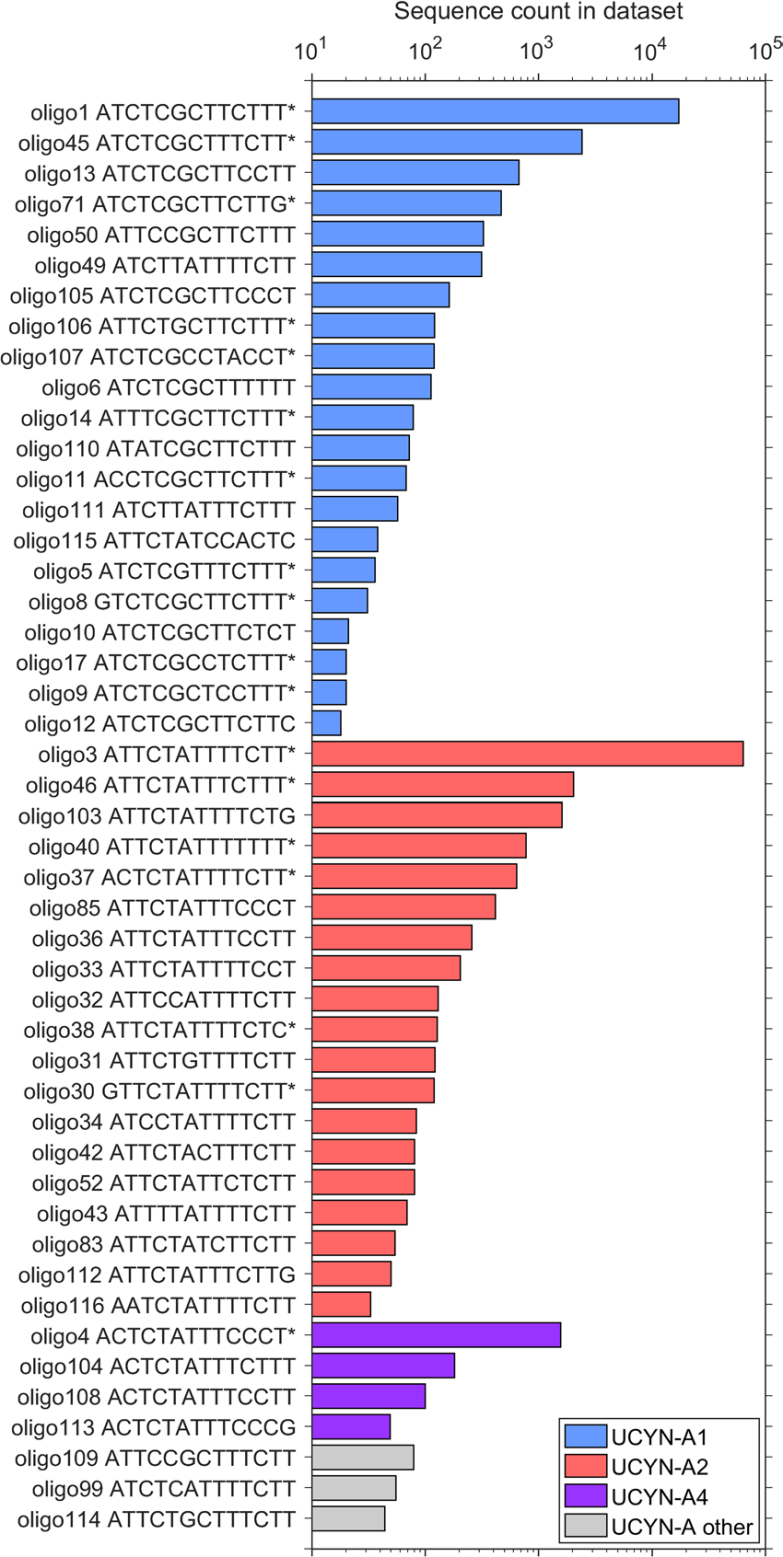
Sequence counts for the 47 UCYN-A oligotypes. Oligotype designation and nucleotide present in each of the 13 high entropy positions are listed. Oligotypes (18 out of 47) with representative sequences that have 100% nucleotide identity to sequences submitted to NCBI’s GenBank database are marked with an asterisk (*). ‘UCYN-A other’ includes oligotypes that did not fall into defined sublineages^14^. *x* axis in logarithmic scale.

Oligotyping analysis resulted in 47 UCYN-A oligotypes, which represented 95163 reads, accounting for 98.4% of all UCYN-A reads analyzed. Fourteen new oligotypes were defined (oligo103‒116). Most oligotypes were phylogenetically affiliated with the sublineages UCYN-A2 (19 out of 47; 70785 sequences or 74.4% of data set) and UCYN-A1 (21 out of 47 oligotypes; 22305 sequences or 23.4% of data set) (Fig. 4, Table S3). Four oligotypes (oligo4, 104, 108, 113; 1895 sequences or 2.0% of data set) clustered with the UCYN-A4 sublineage defined by Farnelid *et al*.^13^. The remaining three oligotypes (oligo99, 109, 114; 178 sequences) did not fall into the sublineages defined to date^17^. However, because they are low in frequency (<80 read counts each), we have not created new clusters for them. All but nine minor oligotypes (oligo6, 11, 36, 37, 85, 104, 109, 111, 114) met the criteria of “convergence” since further decomposition of the oligotypes did not improve the resolution of beta diversity^18^.

**Figure 5:**
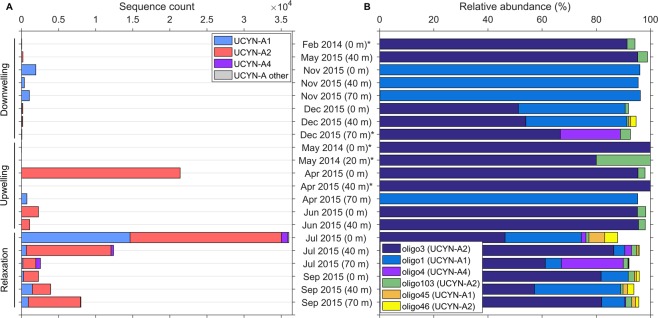
Distribution of UCYN-A sublineages and oligotypes. (**A**) Number of sequences of UCYN-A oligotypes classified by sublineage, and (**B**) relative abundance of the six most abundant oligotypes. ‘UCYN-A other’ includes oligotypes that did not fall into the defined sublineages^14^. Subsampling as indicated in Materials and Methods was not applied in this figure. Samples marked with an asterisk (*) have < 100 sequences.

The UCYN-A *nifH* sequences were by far dominated by oligo3, which is affiliated with the UCYN-A2 sublineage (63905 sequences; 67.2% of data set), and oligo1 affiliated to UCYN-A1 (17152 sequences; 18.0% of data set) (Fig. 4, Table S3). The remaining oligotypes were always less than 2.5% of the library. Oligo3 was 90.3% of the UCYN-A2 oligotypes and accounted for over 50% of the sequences recovered in 17 out of the 21 total samples analyzed (Fig. 5). Oligo1, the second most abundant oligotype, represented 76.9% of the UCYN-A1 oligotypes and was detected in 12 out of 21 samples at relative abundances ranging from 4% to 96%. On average, oligo3 represented 64 ± 36% of the relative abundances of oligotypes in the samples, whereas oligo1 comprised 26 ± 37%. Oligo1 differs from oligo3 in 7 out of 13 entropy positions.

**Figure 6:**
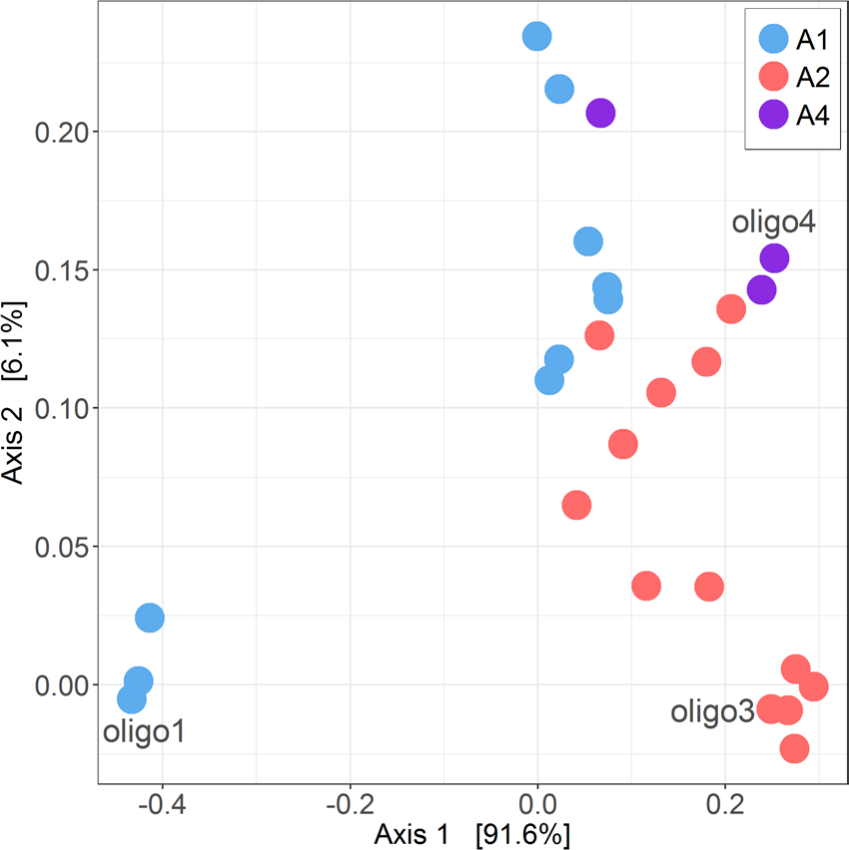
Principal Coordinate Analysis (PCoA) illustrating the Bray-Curtis dissimilarity between UCYN-A oligotypes, which are coloured according to their sublineage (UCYN-A1, -A2 or -A4). The most abundant oligotypes of the data set, oligo1, oligo3 and oligo4 are indicated. Data set was transformed to even sampling depth after subsampling as described in Materials and Methods.

In general under the different hydrographic regimes the oligotype composition was dominated by UCYN-A2, mainly represented by oligo3, reaching the highest sequence absolute abundances during upwelling and relaxation (Fig. 5). Notable exceptions to this include samples from November 2015 (downwelling), and from 70 m depth in April 2015 (upwelling). In these samples, the oligotype composition was dominated by UCYN-A1, mainly represented by oligo1, reaching > 95% of relative abundances. During relaxation conditions, UCYN-A1, mostly oligo1, was also present making up lower relative abundances (4–32%). In general, a larger diversity of oligotypes was found during relaxation since minor oligotypes as oligo4 (UCYN-A4), oligo45 (UCYN-A1), oligo46 (UCYN-A2) and oligo103 (UCYN-A2), were also present in these samples (Table S3). UCYN-A4 oligotypes, mostly oligo4, appeared only at relatively high frequencies in July 2015 (relaxation), representing 2–23% of relative abundance, when UCYN-A2 dominated the oligotype composition. Whereas UCYN-A1 was detected at relatively low frequencies in samples collected at the three hydrographic conditions, UCYN-A2 exhibited higher abundances during upwelling and relaxation. This pattern determines the contrasting locations of their most abundant oligotypes (oligo1 and oligo3, respectively) on the PCoA ordination based on the Bray-Curtis ecological distance between oligotypes from the UCYN-A *nifH* amplicon library (Fig. 6).

## Discussion

### Composition and temporal variability of the diazotroph community: UCYN-A population dynamics

Our findings indicate that the diazotroph community composition in the coastal shelf region on the NW Iberian Peninsula was dominated by UCYN-A and *P. stutzeri*-like taxa (1G). These results support prior reports indicating that the UCYN-A–prymnesiophyte symbiosis is present in these relatively cold (<17 °C) and N-rich waters of the eastern basin of the North Atlantic. Agawin *et al*.^45^ reported the dominance of UCYN-A in a nearby region during a single observation in summer 2009, but in this case UCYN-A represented 95% of *nifH* sequences recovered. Furthermore, UCYN-A were also the most representative cyanobacterial diazotrophs detected by Rees *et al*.^49^ in the temperate waters of the Western English Channel in summer 2006. The relevance of UCYN-A was also reported in the (sub)tropical region of the NE Atlantic^12,50–53^, where regular iron inputs by Saharan dust^54^ promote its growth and N_2_-fixing activity^55^. Similarly, UCYN-A dominated the diazotroph community, together with Proteobacteria, in the N-rich temperate NE American coast of the western basin^56^.

Our results showed an important temporal variability in the diazotroph community composition. Overall, during upwelling and relaxation, affected by enhanced vertical stratification and larger variability in the hydrographic conditions, a similar contribution of a few cyanobacterial and non-cyanobacterial taxa was observed (both UCYN-A and bacteria of clusters 1G and 3). During downwelling, characterized by deeper mixed layers and homogeneous hydrographic conditions, the community composition exhibited higher diversity and lower temporal and vertical variability, being mainly dominated by non-cyanobacterial diazotrophs from clusters 1G and 3.

In the case of upwelling, we observed the predominance of non-cyanobacterial diazotrophs from cluster 3, specifically *Opitutaceae* bacterium-like taxa, in May 2014, and of clusters 1G (Gammaproteobacteria) and 3 in April and June 2015. Nevertheless, we found high UCYN-A relative abundances in the well-lit surface waters of these two samplings. The different stage of the upwelling may induce these *nifH* compositional differences between cruises. In April and June 2015 an intense upwelling of subsurface waters occurred just before sampling, cooling and fertilizing the water column, while in May 2014 we found a more stratified and warmer water column due to the progressive weakening of the intense upwelling pulse that occurred one to two weeks before sampling^57^. The particular dominance of *Opitutaceae* bacterium-like taxa (cluster 3) observed exclusively in May 2014 could correspond to a specific advanced stage of marine bacterioplankton succession after an intense phytoplankton bloom, since different bacterial populations are specialized in the successive decomposition of organic substrates derived from blooms^58^.

The diazotrophic community during summer relaxation condition was characterized by an enhanced contribution of UCYN-A. This observation is consistent with the results obtained by Agawin *et al*.^45^ in a nearby region in summer 2009 under comparable hydrographic conditions. Some differences were observed in the samples collected during the two samplings carried out in relaxation (July and September 2015). In July 2015, the relative abundances of UCYN-A (52 ± 40%) were slightly higher than in September 2015 (27 ± 11%). Although both samplings were characterized by relatively enhanced vertical stratification, nitrate and phosphate concentrations at deep samples were higher in September 2015, due to the upwelling pulse occurring one week before the sampling^57^. The contrasting hydrographic features may have induced the differential *nifH* composition observed between the two cruises.

The highest UCYN-A relative abundances were quantified in stratified surface waters under upwelling and relaxation conditions, coinciding with the highest UCYN-A absolute abundances (via qPCR) and biological N_2_ fixation rates (~0.1 μmol N m^−3^ d^−1^) measured during this survey (reported in Moreira-Coello *et al*.)^57^. In fact, UCYN-A relative and absolute abundances were positively correlated with N_2_ fixation rates (Pearson’s *r* = 0.709, *p* < 0.01 and *r* = 0.534, *p* < 0.05, respectively), consistent with the fact that small diazotrophs (<10 μm), presumably unicellular, were responsible for the diazotrophic activity in this system. This relationship has also been observed in other regions, such as the NE American coast^56^, or the tropical SW Pacific^59^. Therefore, UCYN-A–prymnesiophyte associations may be the main contributors to the diazotrophic activity in this region.

To our knowledge, this is the first study reporting temporal variability of the diazotrophic community composition in a temperate upwelling ecosystem, since most phylogenetic studies targeting the *nifH* gene in marine environments are mainly constrained to (sub)tropical regions. Hence, our study increases the knowledge about temporal dynamics of diazotrophs adding coastal upwelling ecosystems to the previous studies. Similar to our findings, Messer *et al*.^27^ provided evidence of the seasonal shift of diazotroph communities by reporting different assemblages during austral spring (dominance of Cyanobacteria, mainly *Trichodesmium* and UCYN-A) and winter (predominance of Delta- and Gammaproteobacteria) in Australian tropical waters. Church *et al*.^28^ observed a temporally variable diazotroph assemblage in the North Pacific Subtropical Gyre, dominated by Cyanobacteria (*Trichodesmium* and symbionts of diatoms) in summer, and unicellular diazotrophs (UCYN-A) in late winter and spring. In two mesohaline, temperate Danish estuaries, Bentzon-Tilia *et al*.^30^ showed that the diazotroph community varied over a year, alternating between heterotrophic bacteria, UCYN-A and heterocystous cyanobacteria such as *Anabaena*. Turk-Kubo *et al*.^60^ reported the succession of diazotrophs in the Noumea lagoon (New Caledonia, tropical SW Pacific) over a 23-day period during the austral summer, and Robidart *et al*.^26^ demonstrated in the North Pacific Subtropical Gyre that diazotroph communities are highly variable, even at short temporal scales of a few days.

Overall, our results indicate that the diazotrophic community present in the NW Iberian upwelling system is dynamic and responds to the variations of the hydrographic conditions. This is consistent with recent findings reported by Martín-Platero *et al*.^61^ along the Northeast American coast. These authors reported sharp and rapid fluctuations of microbial planktonic OTUs associated with changes in the environmental conditions, suggesting that the high variability in the hydrographic features could provide short-lived but optimal conditions for the growth of different microbial communities.

### Variability and distribution of UCYN-A sublineages

Our oligotyping analysis indicate that UCYN-A2 was by far the most abundant sublineage (74% of the data set), followed by UCYN-A1 (23%) and UCYN-A4 (2%). These UCYN-A clades were mainly represented by oligo3 (67% of data set), oligo1 (18%) and oligo 4 (2%), respectively. These oligotypes were also among the most abundant in the global UCYN-A *nifH* amplicon library analyzed by Turk-Kubo *et al*.^17^, although this database was mainly dominated by oligotypes affiliated to the UCYN-A1 sublineage. This UCYN-A1 prevalence observed in the global data set reflects the fact that most sampling sites were located in oligotrophic open ocean regions of the (sub)tropical Pacific and Atlantic.

Our results provide further evidence that the UCYN-A2 sublineage can be found in nutrient-rich coastal waters. Whereas UCYN-A1 was detected at relatively low frequencies in samples from the three hydrographic conditions, UCYN-A2 exhibited higher sequence counts during upwelling and relaxation. Interestingly, UCYN-A4 appeared only at relatively high frequencies during relaxation in July 2015, when UCYN-A2 dominated the oligotype composition. These results are consistent with previous findings indicating that the most abundant sublineages, UCYN-A1 and UCYN-A2, are globally distributed^12,62^ and co-occur frequently in coastal areas^17,60,63,64^. They are also consistent with previous studies indicating that UCYN-A1 and UCYN-A3 co-occur and dominate in the (sub)tropical open ocean, whereas UCYN-A2, sometimes co-occurring with UCYN-A4, occupy mainly coastal regions^15,17,27,63^. Even though current data suggest that the different UCYN-A sublineages and their hosts probably have different physiological needs (e.g. nutrient requirements), they can co-occur within the same environment, indicating that these ecotypes may under some circumstances share a similar niche^13,15,17^. UCYN-A1 and -A2 are associated with two distinct prymnesiophyte partners, an uncultured unicellular prymnesiophyte (1–3 µm)^14^ and the larger *Braarudosphaera bigelowii* (4–10 µm)^15,62,65^, respectively. The different global distribution and temporal variability of these sublineages may be due to the adaptation of their respective prymnesiophyte hosts to specific environments, and the fact that they go through different life cycle stages^65^. The evolutionary divergence of UCYN-A giving rise to the different ecotypes^66^ could have contributed to their dispersion and growth in contrasting habitats and their ability to overcome environmental changes.

## Conclusions

Our findings show a highly variable diazotroph community shaped by the hydrodynamic forcing of the system, shedding light on the seasonal dynamics of diazotrophs in upwelling regions. The main compositional trait of the local diazotrophic community is the appearance of UCYN-A during upwelling and relaxation conditions. During downwelling, which was characterized by deeper mixed layers and uniform hydrographic conditions, the diazotrophic community composition was vertically more homogeneous and characterized by the dominance of diverse non-cyanobacterial diazotrophs, mostly from clusters 1G (Gammaproteobacteria) and 3 (putative anaerobes). Conversely, upwelling and relaxation periods were characterized by enhanced vertical stratification, a larger variability in the hydrographic features and a more heterogeneous community composition. In these conditions, UCYN-A and non-cyanobacterial diazotrophs from clusters 3 and 1G were dominant. Within UCYN-A phylotype, UCYN-A2 sublineage was by far the most abundant, followed by UCYN-A1 and UCYN-A4. These sublineages were mainly represented by the oligotypes oligo3, oligo1 and oligo 4, respectively, among the most abundant in the global UCYN-A *nifH* library^17^. UCYN-A1 was detected at relatively low frequencies in samples collected during the three hydrographic conditions, while UCYN-A2 showed higher abundances during upwelling and relaxation. Overall, the compositional heterogeneity observed in response to the temporal shifts in environmental factors may reflect the transition of ecological niches of specific diazotrophs, since the community comprises a diverse group of organisms with different autoecology and physiological constraints. In the future, higher temporal resolution approaches focusing on abundance, diversity and expression of *nifH* genes will be required to better understand the role of the short-term variability of hydrodynamic forcing, occurring at scales of a few days in this region^43^, in shaping the diazotrophic community. Furthermore, specific observational and experimental approaches, including nutrient addition assays, should be implemented to shed light on the factors driving the activity and growth of UCYN-A sublineages, as well as to demarcate accurately the environmental conditions that define their ecological niches.

## Materials and Methods

### Hydrographic and environmental sampling

Between February 2014 and December 2015, within the framework of the NICANOR (Nitrogen fixation and diffusive fluxes in the NW Iberian Peninsula) project, nine one-day samplings were carried out on board B/O Lura at station 2 (43.42°N, 8.44°W; depth = 80 m) of the RADIALES time-series project (www.seriestemporales-ieo.net). This station is located in the adjacent shelf off Ría de A Coruña (Golfo Ártabro, NW Iberian Peninsula). On each sampling day, profiles of temperature, salinity and fluorescence were obtained with a CTD (Conductivity-Temperature-Depth) probe SBE25plus (SeaBird Electronics) attached to a rosette of Niskin bottles. Water samples were collected for the determination of inorganic nutrients (NO_3_^−^ and PO_4_^3−^), biological N_2_ fixation, as well as DNA extraction. In 7 out of the 9 surveys, measurements of dissipation rates of turbulent kinetic energy were conducted using a microstructure turbulence profiler (MSS)^67^. The squared Brunt Väisälä frequency (N^2^), a proxy for water column stratification, was computed from the CTD profiles according to the equation:

1 $${N}^{2}=-(\frac{g}{{\rho }_{w}})(\frac{\partial \rho }{\partial z})\,({s}^{-2})$$

where g is the acceleration due to gravity (9.8 m s^−2^), *ρ*_*w*_ is seawater density (1025 kg m^−3^), and *∂ρ/∂z* is the vertical potential density gradient.

The sampling dates were selected to cover contrasting hydrographic conditions in this system over a seasonal cycle, and spanned a period of 20 months. We encountered the main hydrographic features of a temperate coastal region subject to seasonal variability and influenced by upwelling pulses. To simplify the interpretation of the results, we classified the samplings into three hydrographic conditions based on the wind-driven upwelling index (see Fig. 2 in Moreira-Coello *et al*.)^57^ and the hydrographic conditions of the water column (Fig. S5): downwelling, upwelling and relaxation. During downwelling conditions, we sampled winter (February 2014) and autumn (November 2015 and December 2015) mixing, and also a spring transitional period under predominance of downwelling (May 2015). In winter, mixing was characterized by slight haline stratification, whereas in autumn thermohaline mixing dominated. In May 2014, April 2015, and June 2015 we sampled spring-summer upwelling, characterized mainly by enhanced phytoplankton biomass. Relaxation conditions included two summer cruises (July 2015 and September 2015), characterized by the transition between intense upwelling and downwelling events during the summer thermal stratification period. Hydrographic features are described in detail in Moreira-Coello *et al*.^57^.

### DNA Samples Collection and Extraction

We collected a total of 25 samples for DNA. During all samplings except February 2014, when only the sample at 0 m was collected, seawater samples were collected at 0, 40 and 70 m depth (in May 2014 sample at 20 m instead of 70 m). Replicate DNA samples were not used in this study. Seawater was transferred to acid-cleaned carboys using acid-washed silicone tubes and kept in darkness until further processing in the laboratory one-two hours later. Microbial biomass was collected by filtering 7.5–10 L of seawater through a 0.22 µm Sterivex^TM^ filter unit (Millipore) using a peristaltic pump. The filters were preserved with 1.8 mL of lysis buffer (50 mM Tris-HCl pH 8.3, 40 mM EDTA pH 8.0, 0.75 M sucrose) and stored at −80 °C until extraction. DNA was extracted using the PowerWater^®^ DNA Isolation Kit (MO BIO, Laboratories, Inc.), quantified and quality-checked (according to the A_260_/A_280_ ratio) using a spectrophotometer NanoDrop 2000^TM^ (Thermo Fisher Scientific).

### *nifH* gene PCR amplification and amplicon sequencing

The *nifH* gene was amplified in a total of 24 DNA samples (*nifH* was not detected in the surface sample from May 2015) by nested PCR using 2 sets of degenerate primers^68,69^: nifH3 reverse primer (5′-ATR TTR TTN GCN GCR TA-3′) and nifH4 forward primer (5′-TTY TAY GGN AAR GGN GG-3′), nifH1 forward primer (5′-TGY GAY CCN AAR GCN GA-3′) and nifH2 reverse primer (5′-ADN GCC ATC ATY TCN CC-3′). The volume of the PCR reactions was 20 µL: 1 µL DNA template, forward and reverse primers (final concentration 1 µM), MgCl_2_ (final concentration 4 mM), dNTPs mix (final concentration 0.2 mM each), Platinum^®^ Taq DNA Polymerase (ThermoFisher Scientific, final concentration 2.5 U), BSA (final concentration 0.4 mg/mL) and PCR grade water. The first round of amplification used nifH3 and nifH4 primers and the following thermocycling conditions: initial DNA denaturation at 95 °C during 4 min, 35 cycles of denaturation at 95 °C (1 min), annealing at 45 °C (1 min) and extension at 72 °C (30 s), followed by a final extension at 72 °C (10 min) and temperature hold at 15 °C. The second round used the nifH1 and nifH2 primer set, 1 µL of PCR product from the first round as template and the following thermocycling conditions: initial denaturation at 95 °C for 4 min, 30 cycles of denaturation at 95 °C (1 min), annealing at 54 °C (1 min) and extension at 72 °C (30 s), followed by a final extension at 72 °C (10 min) and temperature hold at 15 °C. The reactions were run on a T100^TM^ Thermal Cycler (Bio-Rad) at the Instituto Español de Oceanografía (IEO-A Coruña). The amplified products were purified using PCRExtract Mini Kit (5PRIME) and quantified using a spectrophotometer NanoDrop 2000^TM^ (Thermo Fisher Scientific).

*nifH* amplicon-based sequencing was performed at the facilities of IMGM Laboratories GmbH (Germany) on the Illumina MiSeq^®^ Next Generation Sequencing technology (Illumina Inc.). Prior to sequencing, an additional PCR amplification of 10 cycles with custom barcoded nifH1 and nifH2 primers was carried out. After purification and library preparation from the barcoded PCR products, the paired-end sequencing was performed using the MiSeq^®^ reagent kit with V2 chemistry (500 cycles). The resulting 2x250 base pairs (bp) reads were demultiplexed on the instrument.

### Bioinformatics, phylogenetic and statistical analyses

Paired-end reads were merged, selected by length (300–400 bp) and quality filtered (Q20) using the software package PEAR^70^. Chimera removal and determination of OTU (Operational Taxonomic Unit) clusters at 92% nucleotide identity were performed by using the usearch 6.1 algorithm^71^ in QIIME^72^. We used a 92% cut off to facilitate the graphical representation of the overall diazotroph diversity (Figs 1 and 2). Representative sequences from OTUs with ≥ 10 read counts were imported into ARB software environment^73^ and translated into amino acid sequences. Non-*nifH* sequences, as well as sequences with stop codons or with frameshift errors were excluded from the analysis. Sequences were aligned to a reference alignment in a curated and updated *nifH* database^74^ using HMMER algorithm and the Hidden Markov Model profile Fer4_NifH_fs.hmm^75^. Nucleotide sequences were realigned according to the aligned amino acids in ARB. The phylogenetic affiliation of the translated OTUs into the canonical *nifH* clusters defined by Zehr *et al*.^4^ was performed by BLASTx^47^ using genome-derived sequences as references from the updated and curated *nifH* database^74^. We are aware that the relative abundance of phylotypes in the *nifH* Next Generation Sequencing (NGS) library may be influenced by factors such as DNA extraction efficiency, PCR bias^53,76^ and presence of contaminants^77,78^. In order to test if among our *nifH* sequences there were putative PCR reagent contaminants, we performed a BLASTx comparison between the representative sequences of each OTU and known contaminants^74^. To define a putative contaminant we used a conservative cut off (>92% amino acid identity)^79^. 24 minor OTUs were considered contaminants and removed from further analysis. A total of 581732 translated amino acid sequences were obtained after quality filtering, which represented 1276 OTUs. We also performed a BLASTn analysis^47^ against non-redundant NCBI nucleotide database with the representative sequences of the most abundant OTUs with the objective of identifying and naming them, determining their closest relatives (best BLASTn hit).

A neighbor-joining phylogenetic tree was constructed in ARB using the Kimura correction including the translated amino acid sequences from representative sequences of the most abundant OTUs in the *nifH* NGS library (90 OTUs, >700 sequences each, representing 83.1% of the dataset), as well as their closest relatives (determined via BLASTx). The phylogenetic tree and associated heatmap, which shows the relative abundance of each OTU cluster for all the samples of each hydrographic condition considered separately, were visualized by using the Interactive Tree of Life online tool (iTOL)^80^. The relative abundances were shown per condition to represent the dominance of each OTU within the contrasting hydrographic regimes.

Data were analyzed and visualized by using the R packages phyloseq. 1.20^81^, vegan 2.4^82^ and ggcorrplot 3.4.2^83^. To allow for cross-sample comparisons, a subsampling to the minimum sequencing depth (9534 sequences) followed by the calculation of relative abundance of each OTU was applied. Chao1 richness estimator^84^ and Shannon diversity index^85^ were calculated, and rarefaction curves were generated. Species accumulation curves were built using the Ugland’s method^86^. A Bray-Curtis dissimilarity distance matrix was computed and visualized by Principal Coordinates Analysis (PCoA) ordination to illustrate differences in diazotroph community structure of the samples. Sample group centroids for each hydrographic condition (downwelling, upwelling and relaxation) and confidence ellipses displaying the standard deviation of centroid locations were also represented on PCoA by using betadisper function in vegan R package. A non-parametric PERMutational ANalysis Of VAriance (PERMANOVA) was conducted on the Bray-Curtis distance matrix using the adonis function in vegan R package. We tested the dependence of the distances between samples (derived from their phylogenetic composition) in relation to the factor hydrographic conditions. Bonferroni *post hoc* tests were performed to check *nifH* compositional differences between the samples of the hydrographic conditions defined. Similarity percentages and OTUs contributions to the differences between hydrographic conditions were estimated using the similarity percentage method (SIMPER)^87^ by simper function in vegan R package. According to SIMPER analysis, the OTUs that contributed most to the compositional differences observed between conditions were fitted through significant (*p* < 0.05) vector overlays onto the PCoA ordination by using envfit function in vegan R package.

Raw sequence files have been submitted to the Sequence Read Archive (SRA) at the National Center for Biotechnology Information (NCBI) under SRA Accession SRP149412.

### UCYN-A oligotyping

Oligotyping is a recently described computational method to investigate the diversity of closely related but different microbial organisms, often masked for being clustered into a single OTU, using subtle variations in gene sequences^18^. This technique allows us to define oligotypes, highly refined taxa based on nucleotide positions with high variability (Shannon entropy). We have used this approach, adapted by Turk-Kubo *et al*.^17^, to investigate the UCYN-A diversity and define oligotypes. UCYN-A *nifH* sequences were retrieved from the quality filtered and chimera checked whole *nifH* library at 97% of OTU clustering. A total of 100686 UCYN-A *nifH* sequences were obtained and grouped into 53333 unique sequences (100% nucleotide sequence identity) by using the usearch 6.1 algorithm^71^ in QIIME^72^. All sequences but singletons were imported into ARB^73^, where non-*nifH* sequences, with stop codons or with frameshift errors were removed. Sequences that passed all quality controls (96,712 out of 100,686) were aligned to a reference alignment in a curated and updated *nifH* database^74^ using PyNAST tool^88^. Primer regions were trimmed in the Galaxy platform^89^. Shannon entropy analysis (Fig. S6) and oligotyping were carried out by using the oligotyping pipeline version 2.1^18^. As described by Turk-Kubo *et al*.^17^, 13 nucleotide positions with the highest Shannon entropy were chosen to define oligotypes (42, 48, 75, 78, 93, 99, 102, 147, 150, 218, 244, 247, 265). To reduce noise, each oligotype had to: (1) be present in at least one sample, (2) represent more than 0.1% of the reads for at least one sample, and (3) have a most abundant unique sequence with a minimum sequence count of 10. One sample (May 2014, 40 m) was removed from the analysis because it lacked UCYN-A oligotypes after quality filtering. UCYN-A sequences were not detected in the samples from the 70 m depth from May and June 2015. The total purity score of the analysis was 0.40. UCYN-A oligotype distribution data were also analyzed and visualized using the R package phyloseq 1.20^81^. A subsampling was performed by (a) removing samples that had less than 100 total sequences (16 out of 21 samples remained), and (b) removing oligotypes with <100 sequence counts (26 out of 47 oligotypes remained distributed in 16 samples). A transformation to even sequencing depth was performed, followed by the calculation of a Bray-Curtis dissimilarity distance matrix between the UCYN-A oligotypes detected. Bray-Curtis ecological distances were visualized by Principal Coordinates Analysis (PCoA) according to the UCYN-A sublineages present. Oligotypes were assigned to UCYN-A sublineages, which are defined as in Thompson *et al*.^15^, Farnelid *et al*.^13^ and Turk-Kubo *et al*.^17^.

## Supplementary information

Supplementary information
