# Supplementary material for: Temporal variability of diazotroph community composition in the upwelling region off NW Iberia
Source: Sci Rep. 2019 Mar 6;9:3737. doi: 10.1038/s41598-019-39586-4 (PMC6403370; doi:10.1038/s41598-019-39586-4)
Supplement: Supplementary file 1 — Supplementary information [file 41598_2019_39586_MOESM1_ESM.pdf]

Supplementary Information

Temporal variability of diazotroph community composition in the upwelling region off NW Iberia

Víctor Moreira-Coello[1]*, Beatriz Mouriño-Carballido[1], Emilio Marañón[1], Ana Fernández-Carrera[1], Antonio Bode[2], Eva Sintes[3], Jonathan P. Zehr[4], Kendra Turk-Kubo[4] and Marta M. Varela[2]

[1]Facultade de Ciencias do Mar, Universidade de Vigo, Spain
[2]Instituto Español de Oceanografía, A Coruña, Spain
[3]Instituto Español de Oceanografía, Baleares, Spain
[4]Ocean Sciences Department, University of California, Santa Cruz, California, USA

***Correspondence:**
Víctor Moreira-Coello
vmoreira@uvigo.es

Table S1. Number of sequences obtained per sample after quality filtering and before subsampling to minimum sequencing depth to compute relative abundances.

Sample			Number of sequences
Downwelling	Feb 2014	0 m	18540
	May 2015	40 m	15482
		70 m	18115
	Nov 2015	0 m	18417
		40 m	18103
		70 m	18859
	Dec 2015	0 m	22106
		40 m	20433
		70 m	20193
Upwelling	May 2014	0 m	28126
		20 m	26237
		40 m	9534
	Apr 2015	0 m	33925
		40 m	39060
		70 m	25152
	Jun 2015	0 m	17961
		40 m	36019
		70 m	27050
Relaxation	Jul 2015	0 m	49612
		40 m	29774
		70 m	24011
	Sep 2015	0 m	17520
		40 m	20334
		70 m	27169
		Total	581732

Table S2. Distance matrix between samples based on Bray-Curtis dissimilarity indices (%).

	Feb 2014	May 2015		Nov 2015			Dec 2015			Jul 2015			Sep 2015			May 2014			Apr 2015			Jun 2015		
	0 m	40 m	70 m	0 m	40 m	70 m	0 m	40 m	70 m	0 m	40 m	70 m	0 m	40 m	70 m	0 m	20 m	40 m	0 m	40 m	70 m	0 m	40 m	70 m
	**D1**	**D2**	**D3**	**D4**	**D5**	**D6**	**D7**	**D8**	**D9**	**R1**	**R2**	**R3**	**R4**	**R5**	**R6**	**U1**	**U2**	**U3**	**U4**	**U5**	**U6**	**U7**	**U8**	**U9**
**D1**	0																							
**D2**	0.901	0																						
**D3**	0.877	0.902	0																					
**D4**	0.895	0.633	0.951	0																				
**D5**	0.803	0.887	0.925	0.875	0																			
**D6**	0.832	0.810	0.763	0.749	0.726	0																		
**D7**	0.873	0.771	0.756	0.848	0.887	0.767	0																	
**D8**	0.765	0.859	0.797	0.835	0.784	0.779	0.636	0																
**D9**	0.771	0.799	0.794	0.833	0.849	0.722	0.582	0.542	0															
**R1**	0.997	0.991	0.999	0.944	0.988	0.962	0.993	0.993	0.997	0														
**R2**	0.902	0.916	0.965	0.877	0.833	0.770	0.856	0.875	0.880	0.581	0													
**R3**	0.956	0.880	0.703	0.870	0.921	0.719	0.833	0.883	0.856	0.920	0.819	0												
**R4**	0.892	0.862	0.766	0.859	0.828	0.703	0.755	0.780	0.749	0.916	0.780	0.702	0											
**R5**	0.965	0.880	0.792	0.830	0.929	0.803	0.867	0.902	0.836	0.864	0.772	0.663	0.748	0										
**R6**	0.920	0.923	0.771	0.890	0.886	0.832	0.852	0.835	0.867	0.731	0.611	0.682	0.792	0.673	0									
**U1**	0.998	0.996	0.998	0.999	0.999	0.998	0.995	0.997	0.997	0.907	0.972	0.997	0.998	0.998	0.997	0								
**U2**	0.984	0.987	0.994	0.990	0.978	0.993	0.991	0.976	0.985	0.904	0.935	0.994	0.997	0.996	0.994	0.498	0							
**U3**	0.998	0.998	0.995	0.999	1.000	0.999	0.994	0.991	0.991	0.878	0.959	0.997	0.998	0.999	0.995	0.545	0.506	0						
**U4**	0.962	0.933	0.962	0.929	0.992	0.963	0.935	0.966	0.955	0.312	0.493	0.887	0.867	0.858	0.689	0.997	0.999	0.999	0					
**U5**	0.933	0.925	0.806	0.934	0.958	0.918	0.912	0.943	0.933	1.000	0.981	0.824	0.939	0.884	0.833	0.999	0.990	0.987	0.982	0				
**U6**	0.985	0.942	0.854	0.926	0.952	0.915	0.949	0.948	0.940	0.977	0.953	0.868	0.941	0.868	0.903	0.999	0.998	0.999	0.989	0.935	0			
**U7**	0.882	0.889	0.851	0.866	0.796	0.739	0.878	0.855	0.894	0.909	0.752	0.736	0.571	0.807	0.767	0.998	0.992	0.996	0.842	0.973	0.965	0		
**U8**	0.984	0.971	0.931	0.948	0.984	0.935	0.981	0.947	0.948	0.964	0.930	0.849	0.891	0.894	0.922	0.997	0.990	0.996	0.959	0.988	0.830	0.867	0	
**U9**	0.964	0.948	0.755	0.972	0.948	0.889	0.879	0.924	0.896	0.999	0.957	0.805	0.893	0.823	0.793	1.000	0.998	1.000	0.999	0.796	0.765	0.937	0.902	0

Table S3. Distribution of oligotypes across samples from UCYN-A *nifH* library. Number of sequences are provided for the six most abundant oligotypes: oligo3 ATTCTATTTTCTT (100% nucleotide identity to KM012884), oligo1 ATCTCGCTTCTTT (100% identity to KM012889), oligo45 ATCTCGCTTTCTT (100% identity to LC013499), oligo46 ATTCTATTTCTTT (100% identity to KU183544), oligo103 ATTCTATTTTCTG (99% identity to KU218784) and oligo4 ACTCTATTTCCCT (100% identity to LC013598). Sequence counts for the rest of oligotypes (Other UCYN-A oligotypes) were summed for each UCYN-A sublineage detected, and the number of oligotypes represented (*n*) are indicated at each column. 'UCYN-A other' includes oligotypes that did not fall into the sublineages defined hitherto[14], however, because they are low in frequency (<80 read counts each), we have not created new clusters for them.

Sample			UCYN-A1		UCYN-A2			UCYN-A4	Other UCYN-A oligotypes			
			**oligo1**	**oligo45**	**oligo3**	**oligo46**	**oligo103**	**oligo4**	**A1** *n*=19	**A2** *n*=16	**A4** *n*=3	**A other** *n*=3
**Downwelling**	**Feb 2014**	**0 m**	0	0	32	0	1	0	0	2	0	0
	**May 2015**	**40 m**	0	0	183	0	7	0	0	2	0	0
	**Nov 2015**	**0 m**	1858	0	0	0	0	0	76	0	0	0
		**40 m**	363	0	0	0	0	0	17	0	0	0
		**70 m**	1024	0	1	0	0	0	40	0	0	0
	**Dec 2015**	**0 m**	64	0	83	0	2	0	4	9	0	0
		**40 m**	56	1	81	3	1	0	4	4	0	0
		**70 m**	0	0	36	0	2	12	0	3	1	0
**Relaxation**	**Jul 2015**	**0 m**	10300	2126	16879	1755	401	628	2203	1804	248	151
		**40 m**	493	73	10729	55	230	309	76	407	32	2
		**70 m**	151	4	1565	5	43	586	24	134	42	3
	**Sep 2015**	**0 m**	229	17	1875	26	52	0	17	73	0	3
		**40 m**	1246	66	2241	92	37	0	143	89	0	5
		**70 m**	677	112	6562	97	178	30	115	212	7	14
**Upwelling**	**May 2014**	**0 m**	0	0	7	0	0	0	0	0	0	0
		**20 m**	0	0	4	0	1	0	0	0	0	0
	**Apr 2015**	**0 m**	0	0	20391	0	557	0	1	436	0	0
		**40 m**	0	0	2	0	0	0	0	0	0	0
		**70 m**	691	0	0	0	0	0	34	0	0	0
	**Jun 2015**	**0 m**	0	0	2179	0	70	0	0	41	0	0
		**40 m**	0	0	1055	0	28	0	0	21	0	0
	**Total**		17152	2399	63905	2033	1610	1565	2754	3237	330	178

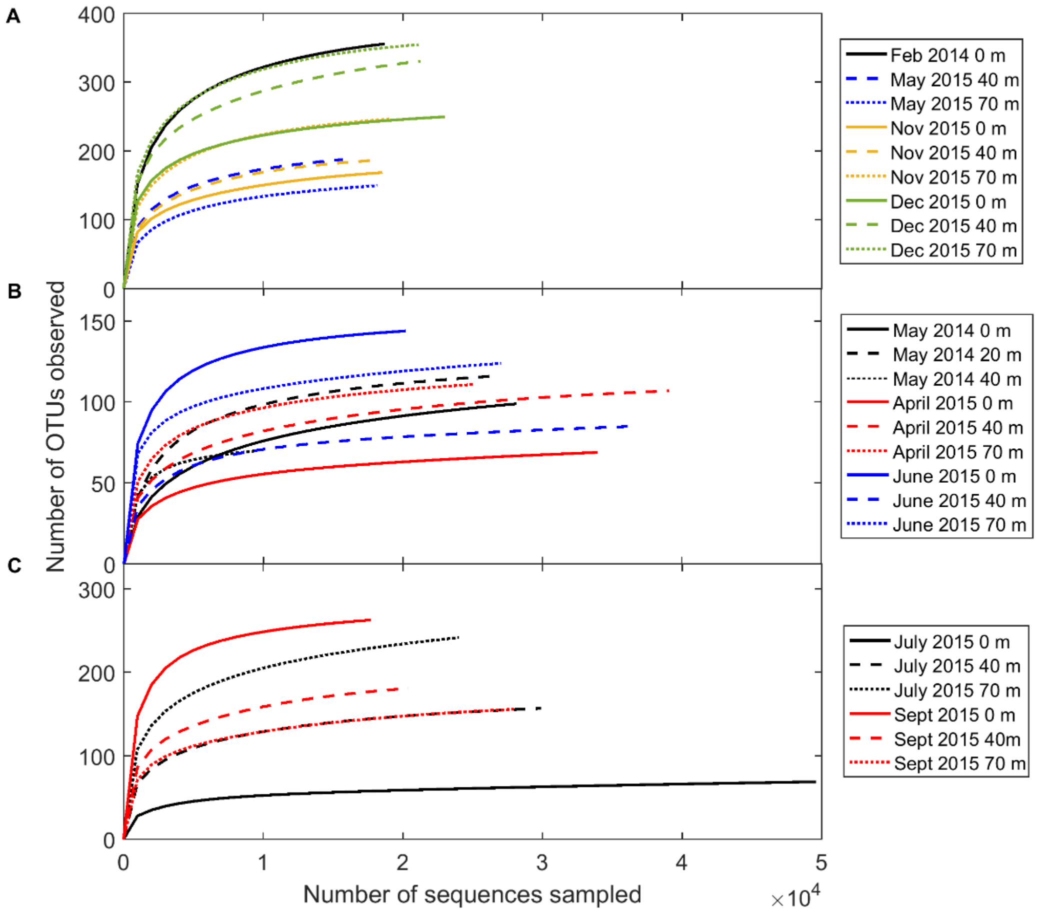

Figure S1. Rarefaction curves of all samples during **(A)** downwelling, **(B)** upwelling, and **(C)** relaxation conditions based on the number of observed OTUs clustered at 92% nucleotide identity cut-off.

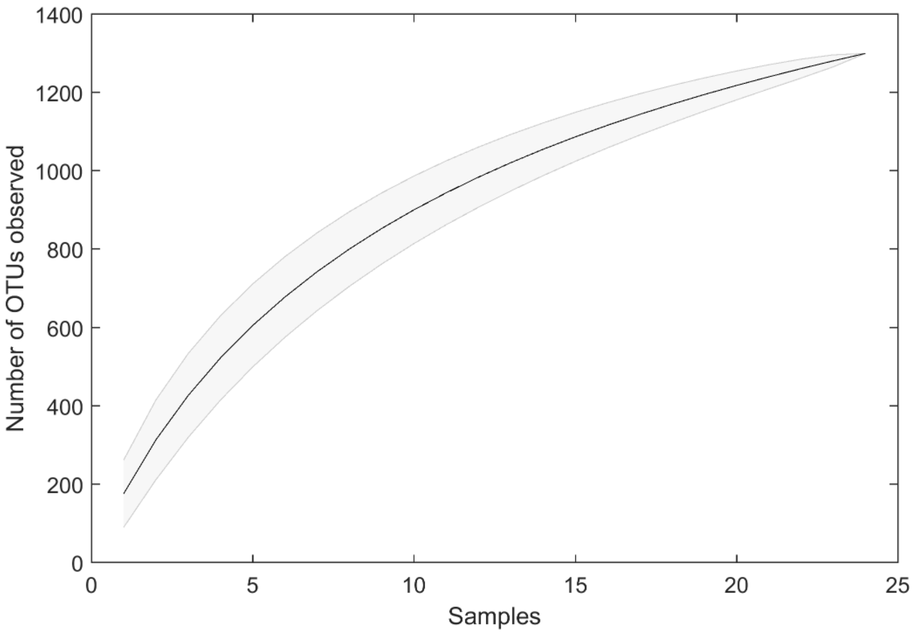

Figure S2. Species accumulation curve constructed using the Ugland´s method[70]. Number of OTUs observed clustered at 92% nucleotide identity for the 24 *nifH* DNA samples collected in the region. Shaded area represents standard deviation.

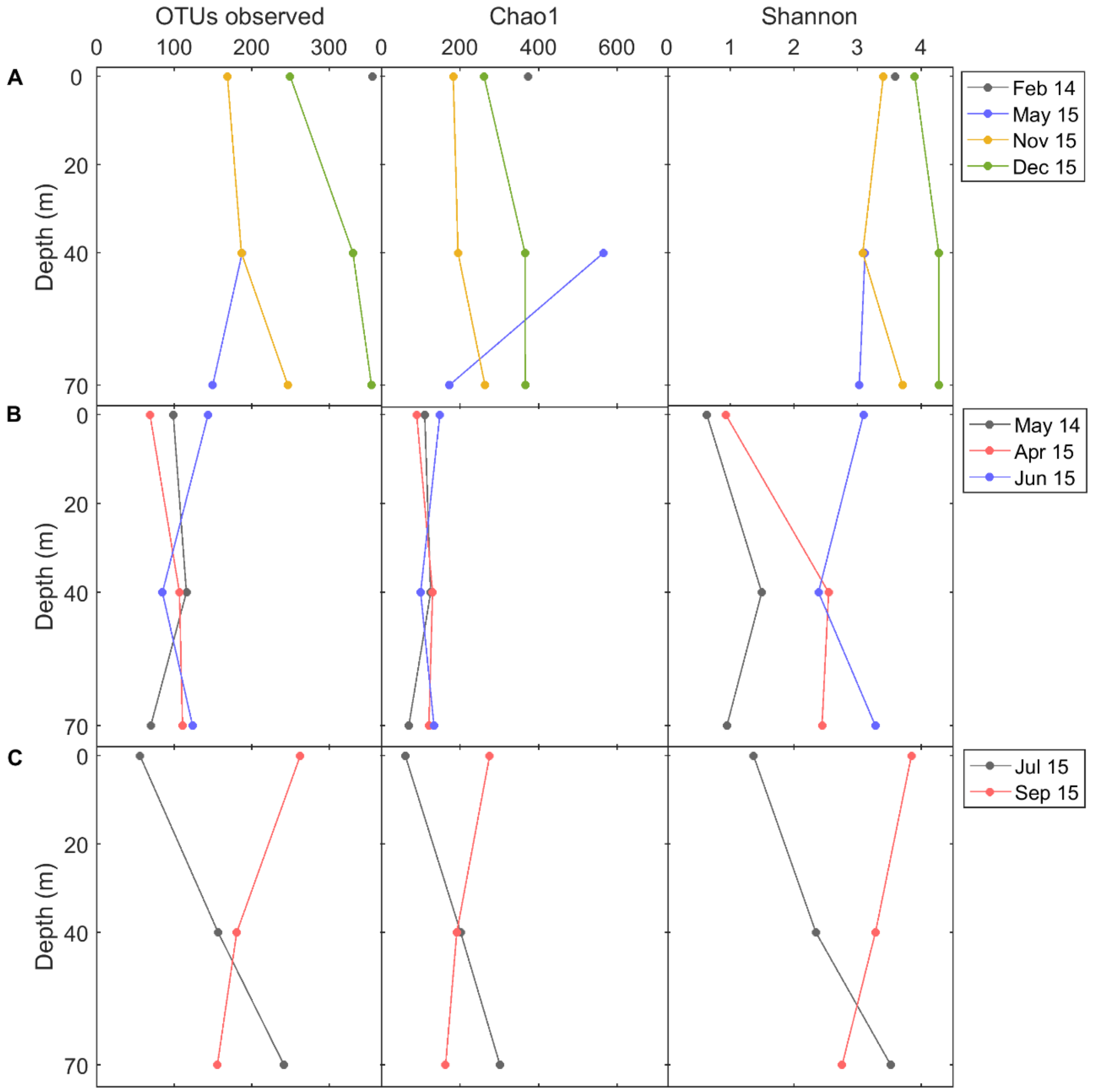

Figure S3. Number of OTUs observed, Chao1 richness estimator[68], and Shannon diversity index[69] at 92% nucleotide identity for subsamples (9534 sequences each) during **(A)** downwelling, **(B)** upwelling, and **(C)** relaxation conditions.

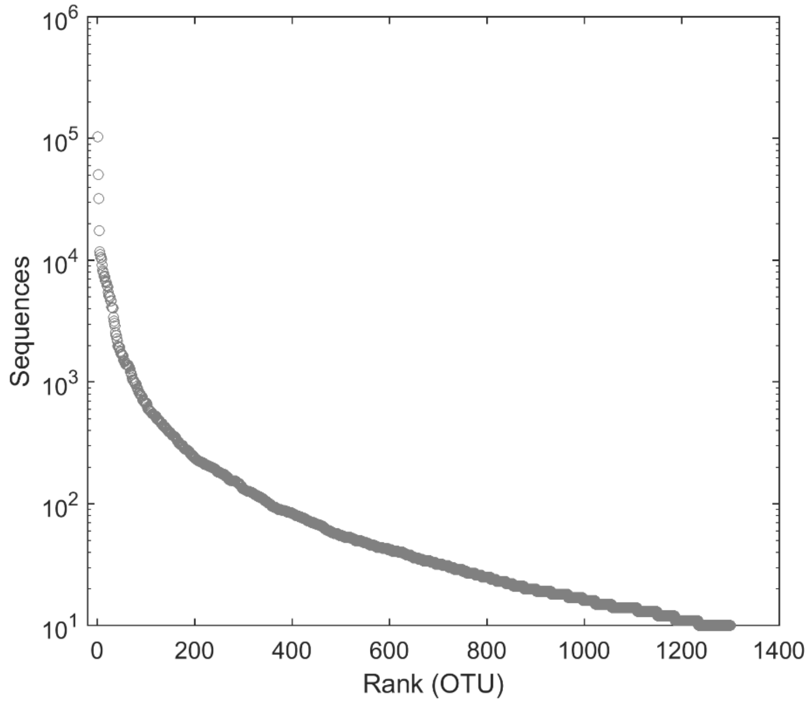

Figure S4. Evenness of the OTUs distribution in the *nifH* library. Number of sequences for each OTU at 92% of nucleotide identity.

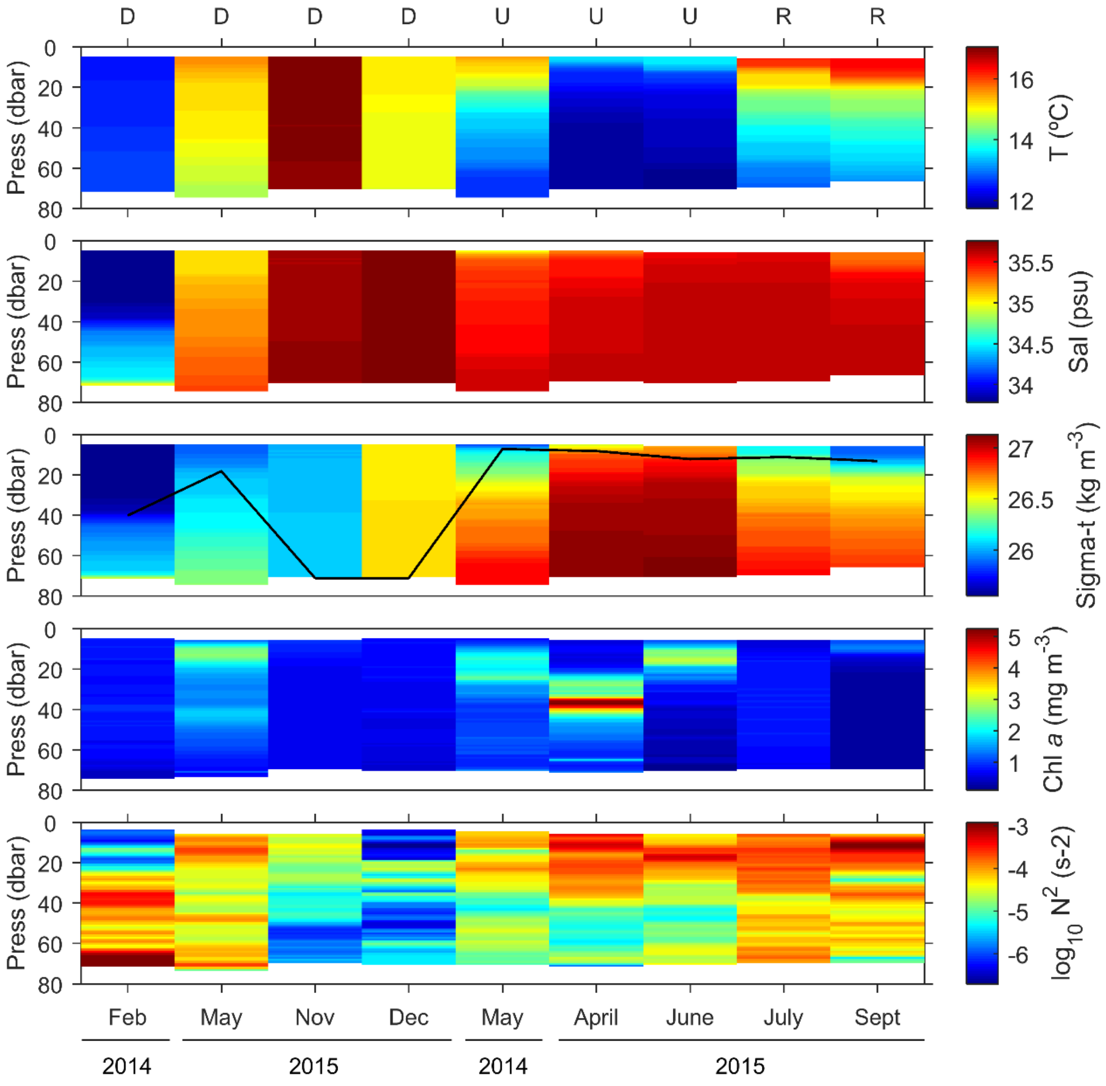

Figure S5. Vertical distribution of temperature (T), salinity (Sal), density (sigma-t), chlorophyll *a* concentration (Chl *a*) and squared Brunt Väisälä frequency (N^2). The black line in the sigma-t panel indicates the mixed layer depth, estimated from an increase in water column density of 0.125 kg m^{-3} relative to surface values. Letters on top panel indicate the hydrographic condition sampled on each cruise (D, downwelling; U, upwelling; and R, relaxation). Chlorophyll *a* concentration was calculated from the calibrated fluorescence sensor (see section Materials and Methods in Moreira-Coello *et al.*[48]).

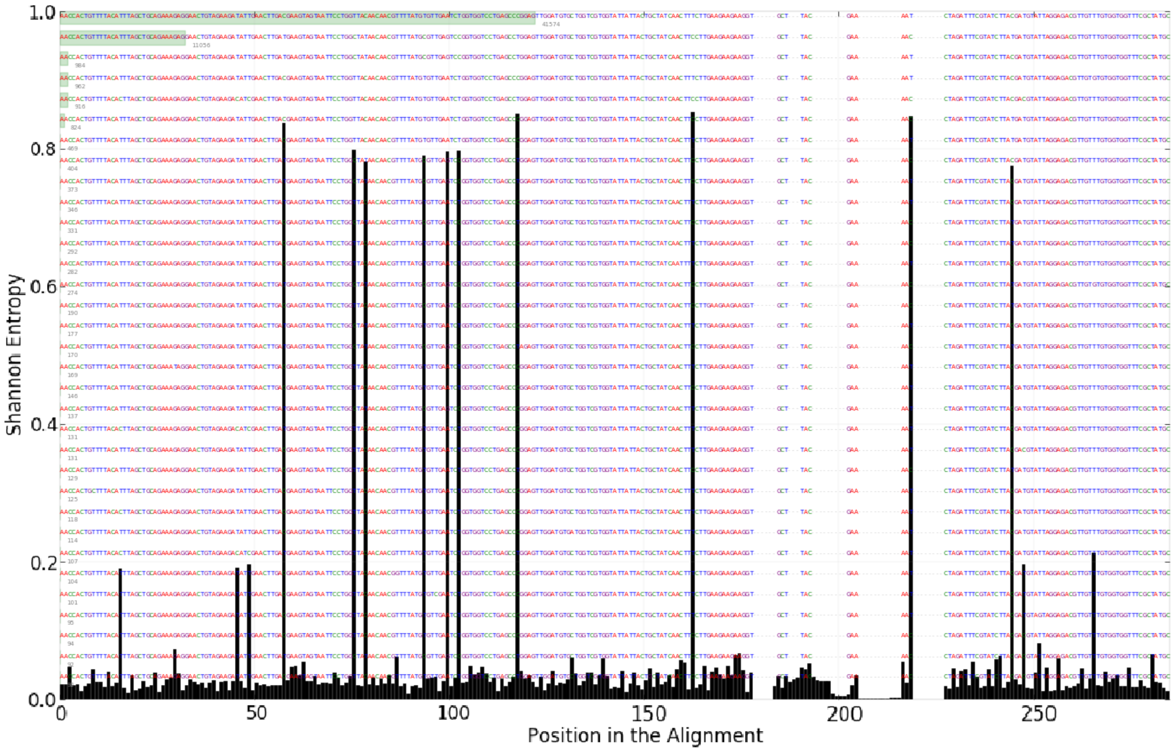

Figure S6. Shannon entropy analysis showing nucleotide positions with highest variability in the data set of UCYN-A *nifH* sequences. Output plot from the oligotyping pipeline (merenlab.org/software/oligotyping/; Eren *et al.*[15]).
